# Nrf2 and Ferroptosis: A New Research Direction for Neurodegenerative Diseases

**DOI:** 10.3389/fnins.2020.00267

**Published:** 2020-04-21

**Authors:** Xiaohua Song, Dingxin Long

**Affiliations:** School of Public Health, University of South China, Hengyang, China

**Keywords:** neurodegenerative diseases, ferroptosis, nuclear factor E2 related factor 2 (Nrf2/NFE2L2), glutathione peroxidase 4 (GPX4), regulation mechanism

## Abstract

Ferroptosis is a kind of regulated cell death (RCD) caused by the redox state disorder of intracellular microenvironment controlled by glutathione (GSH) peroxidase 4 (GPX4), which is inhibited by iron chelators and lipophilic antioxidants. In addition to classical regulatory mechanisms, new regulatory factors for ferroptosis have been discovered in recent years, such as the P53 pathway, the activating transcription factor (ATF)3/4 pathway, Beclin 1 (BECN1) pathway, and some non-coding RNA. Ferroptosis is closely related to cancer treatment, neurodegenerative diseases, ischemia–reperfusion of organ, neurotoxicity, and others, in particular, in the field of neurodegenerative diseases treatment has aroused people’s interest. The nuclear factor E2 related factor 2 (Nrf2/NFE2L2) has been proved to play a key role in neurodegenerative disease treatment and ferroptosis regulation. Ferroptosis promotes the progression of neurodegenerative diseases, while the expression of Nrf2 and its target genes (Ho-1, Nqo-1, and Trx) has been declined with aging; therefore, there is still insufficient evidence for ferroptosis and Nrf2 regulatory networks in the field of neurodegenerative diseases. In this review, we will provide a brief overview of ferroptosis regulatory mechanisms, as well as an emphasis on the mechanism of Nrf2 regulating ferroptosis. We also highlight the role of ferroptosis and Nrf2 during the process of neurodegenerative diseases and investigate a theoretical basis for further research on the relationship between Nrf2 and ferroptosis in the process of neurodegenerative diseases treatment.

## Introduction

Programed cell death is critical to all aspects of mammalian growth and development, homeostatic regulation, and disease control and is closely integrated with other biological processes to sustain life. Ferroptosis is a process that is different from apoptosis, pyroptosis, necroptosis, and other programed cell death (Dixon et al., 2012). In 2018, The International Cell Death Nomenclature Committee defined ferroptosis as a form of regulated cell death (RCD) caused by oxidative alterations in the intracellular microenvironment controlled by glutathione (GSH) peroxidase 4 (GPX4) constitutively, and it can be inhibited by iron chelators and lipophilic antioxidants (Galluzzi et al., 2018). Morphologically, the most important morphological changes of ferroptosis are mitochondria, including mitochondrial contraction, electron-dense mass formation under ultrastructure, reduction or disappearance of mitochondrial cristae, changes in membrane potential, and rupture of mitochondria outer membrane (Vanden Berghe et al., 2014). In addition to the final key morphological changes, the increase of free iron and lipid peroxidation are also important features of ferroptosis, and it is also related to the instability of the plasma membrane, disrupted protein stability, and cytoskeleton rearrangement, leading to the obvious “balloon” phenotype of the cells (Louandre et al., 2013; Gao et al., 2015; Agmon et al., 2018; Dodson et al., 2019), and finally caused cell death. In recent years, with the rapid development of ferroptosis-related research in many fields, it has made a great impact on the fields of cancer therapy, neurodegenerative diseases, ischemia and reperfusion, etc. After the formal concept of ferroptosis, the researchers began to believe that ferroptosis is the main driver of neuronal death in diseases such as Parkinson’s disease (PD), Alzheimer’s disease (AD), and Huntington’s disease (HD) (Guiney et al., 2017; Morris et al., 2018; Mi et al., 2019).

Ferroptosis is characterized by the massive accumulation of fatal intracellular lipid reactive oxygen species (ROS) when the antioxidant capacity of cells decreases (Dixon et al., 2012). Studies have found that ferroptosis is regulated by GPX4 (system Xc-) (Dixon et al., 2012; Friedmann Angeli et al., 2014; Yang et al., 2014), lipid synthesis (Doll et al., 2017; Gao and Jiang, 2018), iron metabolism (Gao et al., 2016; Hou et al., 2016; Hassannia et al., 2019), the mevalonate pathway (Shimada et al., 2016), Nrf2 (or NFE2L2) pathway (Abdalkader et al., 2018; Kerins and Ooi, 2018), and other factors (Luo et al., 2018; Wang et al., 2019c; Ye et al., 2019). Among them, the regulatory effect of transcription factor nuclear factor E2 related factor 2 (Nrf2/NFE2L2) on ferroptosis has attracted our interest. The basic leucine zipper (bZIP) transcription factor Nrf2/NFE2L2 protects cells from oxidative stress by regulating the endogenous antioxidant response pathway. The activity of Nrf2 is strictly regulated by Kelch-like ECH-related protein 1 (Keap1); Keap1 not only passively isolates Nrf2 from the cytoplasm but also plays an active role in targeting Nrf2 for ubiquitination and proteasome degradation (Zhang et al., 2004; Furukawa and Xiong, 2005). Nrf2 can directly or indirectly regulate GPX4 protein content (Hayes and Dinkova-Kostova, 2014; Fan et al., 2017; Dodson et al., 2019; Zhang et al., 2019), intracellular free iron content (Agyeman et al., 2012; Sun et al., 2016b; Shin et al., 2018), mitochondrial function (Merry and Ristow, 2016; Navarro et al., 2017), nicotinamide adenine dinucleotide hydro-phosphoric acid (NAPDH) regeneration (Abdalkader et al., 2018), etc., thereby regulating ferroptosis process. Of note, many studies found that Nrf2 is expressed in the central nervous system (CNS), neurons, astrocytes, leukocytes, and microglia (Tanaka et al., 2011; Dang et al., 2012; Sandberg et al., 2014), and numerous evidence suggests that Nrf2 plays an important role in the development and treatment of neurodegenerative diseases (Wang et al., 2017; Deng et al., 2019; Jang et al., 2019; Pachón-Angona et al., 2019; Wei et al., 2019). Nrf2 has already acted as a well-known target for neurodegenerative disease treatment (de Oliveira et al., 2018; Muhammad et al., 2019; Wang et al., 2019a) and plays a key role in neuronal resistance to oxidative stress and glutamate-induced excitotoxicity and promotes neuronal degeneration and neuronal survival in acute nerve injury. However, in the field of neurodegenerative diseases, there is insufficient research evidence to show that Nrf2 can protect nerve and its related regulatory factors by regulating the mechanism of ferroptosis.

## The Regulation Network Of Ferroptosis

### The Lipid Peroxidation Pathway for Ferroptosis Regulation

**FIGURE 1:**
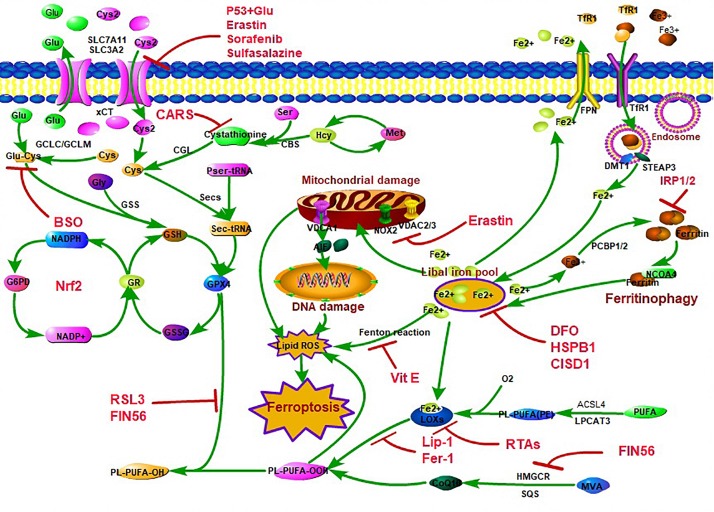
The classic control network of ferroptosis. There are three main characteristics for ferroptosis, including lipid peroxidation, amino acid metabolism disorder, and iron accumulation. The lipid hydroperoxide metabolic pathway is mainly controlled by Acyl-CoA synthetase long-chain family member 4 (ACSL4), lysophosphatidylcholine acyltransferase (LPCAT3), and lipoxygenases (LOXs). ACSL4 catalyzes the attachment of arachidonic acid (AA) or adrenaline (AdA) to produce AA or AdA acyl Co-A derivatives, which is then esterified to phosphatidylethanolamine (PE) (AA-PE and AdA-PE) by lysophosphatidylcholine acyltransferase 3 (LPCAT3). Subsequently, AA-PE and AdA-PE are oxidized by LOXs to produce lipid hydroperoxide, which ultimately leads to ferroptosis. The amino acid metabolism disorder pathway is mainly owing to glutathione (GSH) peroxidase 4 (GPX4) synthesis and function blocking. GPX4 resists iron and oxygen-dependent lipid peroxidation by converting lipid peroxides (L-OOH) to non-toxic lipids and acts as the key enzyme in ferroptosis regulation. GSH is a cofactor and a synthetic substrate for GPX4 and is required for the lipid repair function of GPX4. GPX4 synthesized by GSH requires the pentose–phosphate pathway to supply ATP [through the nicotinamide adenosine dinucleotide hydro-phosphoric acid (NADPH) cycle]. GSH is synthesized by glutamate (Glu), cysteine (Cys), and glycine (Gly) and consists of ATP-dependent glutamate-cysteine ligase (GCL) and GSH synthetase (GSS) through cystine/glutamate reverse transport system xCT [12-channel transmembrane protein transporter vector family 7 member 11 (SLC7A11)/single-channel transmembrane regulatory protein solute carrier family 3 member 2 (SLC3A2)] or sulfur transfer pathway [methionine (Met)–homocysteine (Hcy)–cysteine (Cys) pathway]. When xCT/sulfur transfer pathway is inhibited, the synthesis of GSH and Cys decreasing, which leads to the inhibition of GPX4 synthesis and function to clear LOOH suppression, eventually leading to lipid peroxidation and inducing ferroptosis. The iron accumulation mainly caused by the loss of control of iron transport [membrane iron transporter (FPN), transfer iron protein receptor 1 (TfR1), divalent metal ion transporter 1 (DMT1)] and iron storage [ferritin, degradation *via* the nuclear receptor coactivator 4 (NCOA4)-mediated ferritinophagy pathway], leading to an increase in the concentration of iron in the labile iron pool (LIP) and an increase in reactive oxygen species (ROS) through Fenton reaction/mitochondrial damage/LOX function.

After years of research, people have a certain understanding of the regulatory network of ferroptosis (Figure 1). There are three main regulatory pathways for ferroptosis. Studies have confirmed that ferroptosis selective preferentially oxidizes specific polyphosphorylated phosphatidylethanolamine (PE)-containing polyunsaturated fatty acids (PUFAs), such as arachidonic acid (AA) and epinephrine (Dixon et al., 2015; Kagan et al., 2017), leading to lipid peroxidation in the end, so the lipid peroxidation pathway plays the key role in regulating ferroptosis. Acyl-CoA synthetase long-chain family member 4 (ACSL4), which is a key enzyme that regulates lipid composition, has been shown to contribute to the execution of ferroptosis. During the lipid peroxidation pathway, ACSL4 promotes ferroptosis by producing oxidized PE at the endoplasmic reticulum (ER)-associated oxygenation center, and ACSL4 catalyzes the attachment of AA or adrenaline (AdA) to produce AA or AdA acyl Co-A derivatives, which is then esterified to PE (AA-PE and AdA-PE) by lysophosphatidylcholine acyltransferase 3 (LPCAT3). Subsequently, AA-PE and AdAPE are oxidized by 15-lipoxygenase (15-LOX) to produce lipid hydroperoxide, which ultimately leads to ferroptosis (Doll et al., 2017; Gao and Jiang, 2018), and further research finds that ferroptosis is initiated by the selective and specific peroxidation of sn2-ETE-PE to sn2-15HpETE-PE by 15-LOX/PEPB1 complex (Kagan et al., 2017; Wenzel et al., 2017; Anthonymuthu et al., 2018). There are many regulatory factors for ACSL4 expression; recently, researchers found that special protein 1 (Sp1) was a crucial transcription factor that increased ACSL4 transcription by binding to the ACSL4 promoter region to promote ACSL4 expression to enhance ferroptosis (Li Y. et al., 2019). Lipid peroxidation also happened in p53-mediated ferroptosis. P53 plays a positive role in ferroptosis and identifies coenzyme A (CoA) as a regulator of this cell death process (Leu et al., 2019). Loss of P53 prevents nuclear accumulation of dipeptidyl-peptidase-4 (DPP4) and thus facilitates plasma membrane-associated DPP4-dependent lipid peroxidation, which finally results in ferroptosis (Xie et al., 2017), and p53 suppresses ferroptosis through the induction of cyclin-dependent kinase inhibitor 1A (CDKN1A/p21) expression by reduced accumulation of toxic lipid-ROS (Tarangelo et al., 2018; Kang et al., 2019). Arachidonate 12-lipoxygenase (ALOX12) inactivation diminishes p53-mediated ferroptosis induced by ROS stress (Chu et al., 2019). Interestingly, p53 also plays a negative role in ferroptosis regulation. Spermidine/spermine *N1*-acetyltransferase 1 (SAT1) is a transcription target of p53, SAT1 expression induces lipid peroxidation and sensitizes cells to undergo ferroptosis upon ROS-induced stress, while knockout of SAT1 expression or pharmacologically inhibited ALOX15 partially abrogates p53-mediated ferroptosis (Ou et al., 2016).

### The Glutathione Peroxidase 4 Synthesis and Function-Related Pathway for Ferroptosis Regulation

Amino acid metabolism is also crucial for the regulation of ferroptosis (Angeli et al., 2017). GPX4 is a lipid repair enzyme in our body, and GPX4 was identified as a key regulatory factor in ferroptosis (Dixon et al., 2012; Ingold et al., 2018; Forcina and Dixon, 2019). GPX4 resists iron and oxygen-dependent lipid peroxidation by converting lipid peroxides (L-OOH) to non-toxic lipids (Ursini et al., 1985). GSH is a cofactor and a synthetic substrate for GPX4, and it is required for the lipid repair function of GPX4 (Feng and Stockwell, 2018). GSH is an essential intracellular antioxidant synthesized by glutamate, cysteine, and glycine, which is composed of ATP-dependent cytoplasmic enzymes glutamate-cysteine ligase (GCL) and GSH synthetase (GSS) (Stockwell et al., 2017) and cystine/glutamate reverse transport system Xc- [or xCT/12 channel transmembrane protein transport protein carrier family 7 member 11 (SLC7A11) mediates the uptake of cystine to exchange the output of glutamate] was identified as the pivotal regulator for GSH synthesis (Hao et al., 2018). Inhibition of system Xc- led to the depletion of cysteine, lacking GSH synthetic substrate, and then impaired the function of antioxidant enzyme GPX4 (Friedmann Angeli et al., 2014; Yang et al., 2014), which finally caused an imbalance of homeostatic oxygen homeostasis, leading to ferroptosis (Dixon et al., 2012). Genomic disruption of xCT *via* CRISPR-Cas9 proved that the cystine/glutamate exchanger xCT is essential for amino acid and redox homeostasis in the ferroptosis process (Daher et al., 2019). During the GPX4 synthesis and function-related pathway for ferroptosis regulation, p53 can enhance ferroptosis by inhibiting the expression of SLC7A11 (Jiang et al., 2015; Kang et al., 2019). The histone deubiquitinating enzyme breast cancer 1(BRCA1)-associated protein 1 (BAP1) also inhibits SLC7A11 by reducing H2A ubiquitination (H2Aub) on the SLC7A11 promoter (Zhang et al., 2019). Cytokine signaling 1 (SOCS1) is required for p53 activation and the regulation of cellular senescence, and SOCS1 is sufficient to regulate the expression of p53 target genes and sensitized cells to ferroptosis by reducing the expression of the cystine transporter SLC7A11 and the levels of GSH (Saint-Germain et al., 2017). Histone H2B on lysine 120 (H2Bub1) is an epigenetic mark generally associated with transcriptional activation, and H2Bub1 epigenetically activates the expression of SLC7A11, but p53 decreases H2Bub1 occupancy on the SLC7A11 gene regulatory region and represses the expression of SLC7A11 during ferroptosis process (Wang et al., 2019f). Recently, the OTU domain, ubiquitin aldehyde binding 1 (OTUB1) was demonstrated as a key factor in modulating SLC7A11 stability. OTUB1 directly interacted with and stabilized SLC7A11; conversely, OTUB1 knockdown diminished SLC7A11 levels, finally inducing ferroptosis (Liu et al., 2019). Activating transcription factor (ATF)3 is a member of the ATF/cAMP response element binding protein (CREB) family of transcription factors, and its expression is rapidly induced by a wide range of cellular stresses, including DNA damage, oxidative stress, and cell injury. ATF3 achieved this activity through binding to the SLC7A11 promoter and repressing SLC7A11 expression in a p53-independent manner (Wang et al., 2019b). ATP-binding cassette, sub-family C (CFTR/MRP), member 1 (ABCC1)/multidrug resistance protein 1 (MRP1) was confirmed to mediate GSH efflux from the cell (Cole, 2014). High levels of MRP1 expression could mediate GSH efflux, thus affecting the synthesis of GPX4, and promote collateral sensitivity to ferroptosis-inducing agents in the end (Cao et al., 2019). When investigated, ferroptosis in CD8 + T cells that is activated by cancer immunotherapy, the interferon-gamma (IFNγ), downregulates the expression of SLC3A2 and SLC7A11, impairs the uptake of cystine by tumor cells, and as a consequence, promotes tumor cell lipid peroxidation and ferroptosis (Wang et al., 2019d). Therefore, the stability of SLC7A11 plays a crucial role in GPX4 synthesis and finally regulates ferroptosis.

The function of GPX4 is equally important for the regulation of ferroptosis. ATF4 is a 351-amino acid cAMP-response element binding protein that belongs to the CREB-2 family of proteins. ATF4 resulted in the induction of heat shock protein 70 family protein 5 (HSPA5), which in turn bound GPX4 and protected against GPX4 protein degradation and subsequent lipid peroxidation (Zhu et al., 2017), and GSH-specific gamma-glutamylcyclotransferase 1 (CHAC1) degradation of GSH enhanced ferroptosis through GCN2-eIF2α-ATF4-GPX4 pathway (Chen et al., 2017). Selenium (Se) is an essential micronutrient associated with a broad array of health-promoting effects. Recently, Se was confirmed as a necessary micronutrient in the function of GPX4. When Se in GPX4 was replaced by sulfur, GPX4 loses its ability to inhibit ferroptosis and caused death in a mouse model (Ingold et al., 2018). Remarkably, pharmacological Se supplementation effectively inhibits GPX4-dependent ferroptotic death as well as cell death induced by excitotoxicity or endoplasmic reticulum (ER) stress, which is GPX4 independent (Alim et al., 2019). Collectively, GPX4 synthesis and the function-related pathway are essential in ferroptosis regulation.

### The Iron Metabolism-Related Pathway for Ferroptosis Regulation

Iron is a redox-active metal that can be engaged in free radical formation and propagation of lipid peroxidation. Therefore, the levels of iron can increase the sensitivity for ferroptosis (Hassannia et al., 2019). Iron is also necessary for mitochondrial function, synaptic plasticity, and cognitive function development. Excessive intracellular iron accelerates senescence by destroying DNA and blocking the genome repair system, and such a process was defined as the occurrence of ferritin aging (Sfera et al., 2018). When iron homeostasis in the body is out of balance, autophagy of ferritin (namely, ferritinophagy) mediated by nuclear receptor coactivator 4 (NCOA4) releases iron bounds to ferritin (Kruer, 2013; Dowdle et al., 2014; Mancias et al., 2014; Gao et al., 2016) or an abnormal increase in labile iron pools (LIP) through dysregulation of transferrin and transferrin receptors (iron from the extracellular environment) (Gao et al., 2016; Hou et al., 2016). Then through the Fenton reaction [Fenton’s chemistry refers to a series of reactions between peroxides and divalent ferrous salts, producing oxygen-centered free radicals (Winterbourn, 1995)], producing hydroxyl and peroxy radicals, then extracts oxygen atoms from PUFA diallyl carbon and induces PUFA-PLs peroxidation (Gaschler and Stockwell, 2017), and finally induced ferroptosis. Heme oxygenase-1 (HO-1/HMOX1) is a cytoprotective enzyme induced in response to cellular stress. HO-1 mainly catalyzes the degradation of heme to biliverdin, carbon monoxide (CO), and Fe2^+^, and then increasing LIP to enhance ferroptosis (Adedoyin et al., 2018; Chang et al., 2018).

Mitochondria also make an important impact on regulating iron homeostasis. The NEET (gene symbol, 2 iron, 2 sulfur cluster binding protein. Ordered locus name: At5g51720) proteins mitoNEET {encoded by CDGSH [gene sequence, the iron-sulfur (2Fe-2S) binding motif; the conserved sequence C-X-C-X2-(S/T)-X3-P-X-C-D-G-(S/A/T)-H is a defining feature of this unique family] iron sulfur domain 1 (CISD1)} is an outer mitochondrial membrane protein essential for sensing and regulation of iron and ROS homeostasis (Yuan et al., 2016; Mittler et al., 2018). MitoNEET receives its clusters from the mitochondrion and transfers them to acceptor proteins and limits mitochondrial iron uptake from LIP and therefore suppresses ferroptosis (Yuan et al., 2016). MitoNEET and NAF-1 [encoded by CDGSH iron sulfur domain 2 (CISD2)] interact together by transferring the 2F2-2S cluster to maintain the levels of iron in the mitochondria (Karmi et al., 2017). Voltage-dependent anion channel 1 (VDAC1) is a crucial player in the cross-talk between the mitochondria and the cytosol, and it is regulated by mitoNEET; mitoNEET gates VDAC1 when mitoNEET is oxidized. Further study found that pharmacological inhibition of VDAC1 prevents mitoNEET-dependent mitochondrial iron accumulation *in situ* (Lipper et al., 2019).

### Some News Finding for Ferroptosis Regulation

High-mobility group box 1 (HMGB1) is a nuclear protein that plays a fundamental role in the regulation of DNA-associated events such as DNA repair, transcription, and replication. A recent study found that HMGB1 is a novel regulator of ferroptosis *via* the Ras-c-Jun N-terminal kinase (JNK)/p38 pathway (Ye et al., 2019), and further research found that HMGB1 is a damage-associated molecular pattern (DAMP) molecule released by ferroptotic cells in an autophagy-dependent manner (Wen et al., 2019). Non-coding RNA has also been shown to be involved in regulating ferroptosis in recent years. MicroRNA-137 plays a novel and indispensable role in ferroptosis by inhibiting glutaminolysis (Luo et al., 2018), and at the same time, microRNA-9 was demonstrated regulating ferroptosis by targeting glutamic-oxaloacetic transaminase 1 (GOT1) (Zhang K. et al., 2018). Long non-coding RNA LINC00336 served as an endogenous sponge of microRNA-6852 (MIR6852) to regulate the expression of cystathionine-β-synthase (CBS), and MIR6852 directly binds to LINC0036 and serves as a negative upstream regulator of CBS-mediated ferroptosis inhibition (Wang et al., 2019c).

### Research Progress of Nuclear Factor E2 Related Factor 2 on Ferroptosis Regulation

**FIGURE 2:**
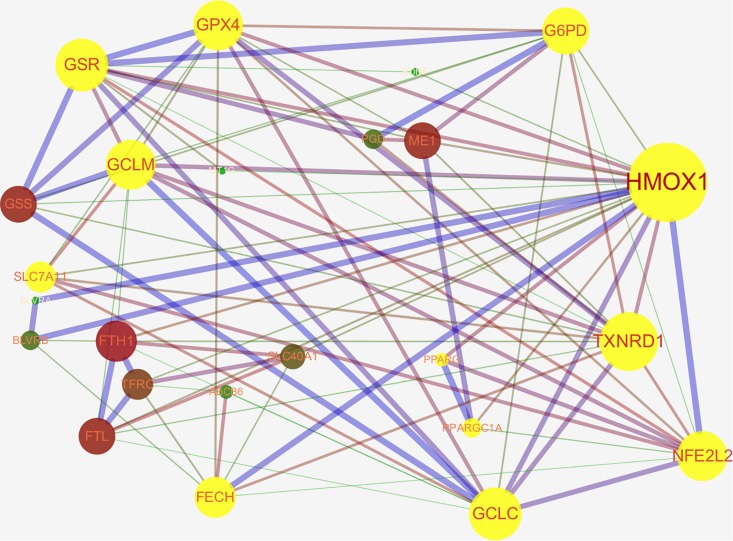
The networks of ferroptosis-related proteins targeted by nuclear factor E2 related factor 2 (Nrf2/NFE2L2) (Protein interaction network from STRING database: https://string-db.org/cgi/input.pl and edited by cytospace3.7.1 software). Nrf2 can regulate ferroptosis directly through GPX4 synthesis-related enzyme [glucose-6-phosphate dehydrogenase (G6PD), glutathione (GSH) reductase (GSR), GSH peroxidase 4 (GPX4), glutamate-cysteine ligase modifier subunit (GCLM), 12-channel transmembrane protein transporter vector family 7 member 11 (SLC7A11), glutamate-cysteine ligase catalytic subunit (GCLC), thioredoxin reductase 1 (TXNRD1)] or through the peroxisome proliferator-activated receptor gamma (PPARγ) pathway. Nrf2 can indirectly regulate intracellular iron concentration *via* Nrf2-heme oxygenase 1 (HMOX1)-iron regulatory-related protein axis [biliverdin reductase A/B (BLVRA/B), ferritin heavy chain 1 (FTH1), transferrin receptor 1 (TFRC), recombinant ferrochelatase (FECH), ferroportin (FPN1/SLC40A11)] to regulate ferroptosis.

Nrf2 is a well-known transcription factor that plays a key role in antioxidation. Downstream genes of Nrf2 include NAD(P)H quinone oxidoreductase 1, HO-1, solute carrier family 7 membrane 11 (SLC7A11/xCT), NAD(P)H quinone oxidoreductase 1, thioredoxin 1, phase II detoxifying enzymes (e.g., GSH S-transferase, UDP-glucuronosyltransferase, GPX4, GSH reductase, and glutamate-cysteine ligase subunits; GCLc and GCLm), and several multidrug resistance-associated transporters (Furfaro et al., 2016; Tonelli et al., 2018). Thus, Nrf2 is considered to be an important regulatory factor for ferroptosis (Abdalkader et al., 2018). The activity of Nrf2 is rigorously regulated by Keap1. Keap1 not only passively isolates Nrf2 from the cytoplasm but also plays an active role in targeting Nrf2 for ubiquitination and proteasomal degradation (Zhang et al., 2004; Furukawa and Xiong, 2005). Under normoxic conditions, Nrf2 binds to Keap1 and continues to be inactivated by ubiquitination and degradation in the proteasome (Lu et al., 2017). Once the body is in oxidative stress, or if there are a large number of electrophiles or cytotoxic agents, Nrf2 is released from the Keap1 binding site and rapidly transferred to the nucleus, subsequently interacting with the antioxidant response element (ARE) in the promoter region of the target gene and then activates the transcriptional pathway to balance oxidative stress and maintain cellular redox homeostasis (Zhang, 2006). Ferroptosis-related genes that are transcriptionally regulated by Nrf2 have been reported, these include genes for GSH regulation (synthesis, cysteine supply *via* SLC7A11, GSH reductase, GPX4), NADPH regeneration which is critical for GPX4 activity [glucose-6-phosphate dehydrogenase (G6PD), phosphogluconate dehydrogenase, malic enzyme], and iron regulation (including iron export and storage, heme synthesis, and catabolism) (Abdalkader et al., 2018; Kerins and Ooi, 2018). We found that Nrf2 can directly or indirectly regulate ferroptosis-related proteins through the STRING database website and Cytoscape 3.7.1 software analysis (Figure 2). In the protein–protein interaction (PPI) network, we obtained that Nrf2 regulates ferroptosis primarily by directly affecting the synthesis and function of GPX4 and the peroxisome proliferator-activated receptor gamma (PPARγ) pathway, and we hypothesize that the regulation of intracellular iron concentration by Nrf2 is mainly through the Nrf2–HO-1 axis. HO-1 is a stress-inducible protein with potential anti-inflammatory and antioxidant properties. HO-1 can metabolize heme to biliverdin, iron, and CO (Kweider et al., 2013). Although many studies have confirmed that Nrf2–HO-1 pathway can regulate intracellular iron concentration (LIP) (Chillappagari et al., 2014; Tomiotto-Pellissier et al., 2018; Cataneo et al., 2019; Selvakumar et al., 2019), there is still no direct evidence to prove our hypothesis that intracellular iron concentration is mainly regulated by the Nrf2–HO-1 pathway. Further research is needed to confirm this hypothesis.

Nrf2 regulates ferroptosis in many ways, and the relationship between Nrf2 and the key pathways for ferroptosis regulation has already attracted attention (Table 1). When cells are exposed to ferroptosis inducers, the SQSTM1/p62–Keap1–Nrf2–AKR1C (metal-binding protein MT-1G) pathway is activated, and then activating transcription of quinone oxidoreductase-1, HO-1, and ferritin heavy chain-1, reducing the sensitivity of ferroptosis in the end (Sun et al., 2016b). Nrf2 can directly or indirectly regulate GPX4 expression and function. The gene responsible for the proteins that encode GSH synthesis, including SLC7A11 (system Xc-), GCLC/GLCM and GSS, GPX4, etc., and oxidoreductases that use GSH and NADPH to reduce oxidative substrates, such as GSH-S-transferase p1 (GSTP1) and α1 (GSTA1), peroxidase 1 (PRDX1) and 6 (PRDX6), and thioredoxin reductase (TXNRD1) are all targets of Nrf2 (Hayes and Dinkova-Kostova, 2014). Nrf2 directly binds to ARE sequence of SLC7A11 subunit promoter and then promotes the expression of SLC7A11 (Carpi-Santos and Calaza, 2018). Nrf2 overexpression or Keap1 knockdown can increase SLC7A11 expression, while inhibition of Nrf2 expression or overexpression of Keap1 decreases SLC7A11 protein expression levels, thereby altering the sensitivity to ferroptosis (Fan et al., 2017; Shin et al., 2017). However, recent research has indicated that PUFAs promoted cystine uptake in placental cells by inducing xCT/SLC7A11 expression and Nrf2 did not contribute to upregulation of xCT/SLC7A11 by PUFAs (Ono et al., 2019). And an H2A deubiquitinase, tumor suppressor BAP1-mediated SLC7A11 inhibition, does not require Nrf2 and ATF4 transcription factors but represses SLC7A11 expression by reducing H2A ubiquitination (H2Aub) on the SLC7A11 promoter (Zhang et al., 2019). When Keap1 has been inhibited, the activity of Nrf2 increases, resulting in upregulation of the ATP-binding cassette (ABC)-family transporter multidrug resistance protein (MRP1), which prevents GSH efflux from the cell and strongly inhibits ferroptosis (Cao et al., 2019). Therefore, we speculate that Nrf2 partially targets SLC7A11 to regulate GPX4 synthesis and function and thereby regulates ferroptosis.

**TABLE 1:** NRF2 target genes are involved in regulating ferroptsis.

**Gene**	**Protein Name**	**Function**	**Regulation of Ferroptosis**	**References**
**Iron Homeostasis-Related**				
HMOX1 (HO-1)	Heme oxygenase 1	Metabolizes heme to biliverdin, iron (Fe^2+^) and carbon monoxide (CO)	Cellular iron availability, labile iron pools (LIP) homeostasis	Kweider et al., 2013; Chang et al., 2018; Hassannia et al., 2018; Hassannia et al., 2019
TFRC	Transferrin receptor	Imports iron into cells	Cellular transferrin-iron uptake, labile iron pools (LIP) homeostasis	Gao et al., 2016; Li et al., 2017; Fillebeen et al., 2019
FTH1	Ferritin heavy chain 1	Stores iron in a soluble, non-toxic, readily available form, subunit of ferritin	Intracellular iron storage protein, labile iron pools (LIP) homeostasis	Andrews and Schmidt, 2007; Bogdan et al., 2016; Sun et al., 2016b; Dodson et al., 2019
FTL	Ferritin light chain			
SLC40A1 (FPN1)	Ferroportin	External non-heme iron intake, exports excess iron from cells	Cellular iron exporter, labile iron pools (LIP) homeostasis	Theurl et al., 2016; Anderson and Frazer, 2017; Brissot et al., 2018
BLVRA/B	Biliverdin reductase-A/B	Converts biliverdin-IX-alpha into bilirubin-IX-alpha	Eliminates by-products of heme metabolism, regulate heme-iron	Sponholz et al., 2012; Zhang Y. et al., 2018
SLC48A1 (HRG1)	Heme responsive gene 1	Heme transporter, recycles heme-iron	Mobilizes heme to cytoplasm, recycles heme-iron	Rajagopal et al., 2008; Campbell et al., 2013; White et al., 2013; Zhang J. et al., 2018
ABCB6	ATP-binding cassette subfamily B member 6	Exports and imports heme and its precursors across the plasma membrane, and outer mitochondrial membrane, respectively	Regulate heme-iron homeostasis	Helias et al., 2012; Boswell-Casteel et al., 2017
FECH	Ferrochelatase	Catalyzes the insertion of Fe2+ ion into protoporphyrin IX	Heme biosynthesis	Basavarajappa et al., 2017; Mirmiran et al., 2019
**GPX4 Synthesis-Related**				
SLC7A11	Solute carrier family 7 member 11	Subunit of system Xc- to import cystine in the cell	Regulation of cysteine and glutamate required for GPX4 synthesis	Lang et al., 2019; Liu et al., 2019; Wang et al., 2019d
GCLM	Glutamate-cysteine ligase modifier subunit	Enzyme involved in GSH synthesis (modifier subunit)	Glutathione (GSH) synthesis	Bea et al., 2003; Yang et al., 2014; Lian et al., 2018
GCLC	Glutamate-cysteine ligase catalytic subunit	Enzyme involved in GSH synthesis (catalytic subunit)		
GSS	Glutathione synthetase	Catalyzes glutathione production from L-γ-glutamyl-L-cysteine	Glutathione (GSH) synthesis	Njålsson, 2005; Djulbegovic and Uversky, 2019; Li F. et al., 2019
GSR	Glutathione reductase	Catalyzes GSSG reduction to GSH by using NADPH as a reducing cofactor	Catalyzes glutathione disulfide (GSSG) reduction to glutathione (GSH)	Hoffmann et al., 2017; Kim et al., 2019
TXNRD1	Thioredoxin reductase-1	Reduces thioredoxin-1 (Trx1) disulfide and supply ATP	Fuels glutathione (GSH) synthesis	Prigge et al., 2017; Ouyang et al., 2018
**Lipid Peroxidation-Related**				
GPX4	Glutathione peroxidase 4	Reduces membrane phospholipid hydroperoxides	Reduces phospholipid hydroperoxide	Yang et al., 2014; Doll et al., 2019; Gao et al., 2019
SCD1	Stearoyl-CoA Desaturase 1	Lipid synthesis	Monounsaturated fatty acid synthesis, decreases CoQ_10_	Huang et al., 2010; Carbone and Melino, 2019; Tesfay et al., 2019
SHP (NR0B2)	Small heterodimer partner	Lipid metabolism	Unknown; lack of direct evidence	Huang et al., 2010; Dodson et al., 2019
PPAR-γ	Peroxisome proliferator-activated receptor gamma	Lipid uptake	Unknown; lack of direct evidence	Huang et al., 2010; Abdalkader et al., 2018
**Others**				
MT1G	Metallothionein-1G	Protection against heavy metals and oxidative injury	Through MT1G-Nrf2 pathway/MT1G-P53-P21 pathway	Sun et al., 2016a; Wang et al., 2019e
G6PD	Glucose-6-phosphate dehydrogenase	Produces ribose and nicotinamide adenine dinucleotide phosphate (NADPH) *via* the pentose–phosphate pathway (PPP).	NADPH regeneration	Dodson et al., 2019; Yang et al., 2019
miR-7	MicroRNA-7	Negatively control gene expression by binding to their target sequences in the 3’-UTR of mRNAs	miR-7-Keap1-Nrf2-HO-1/GCLM pathway	Kabaria et al., 2015

*NRF2, nuclear factor E2 related factor 2.*

Among the regulatory factors known to ferroptosis, the light and heavy chains (FTL/FTH1) of the key iron storage protein ferritin in the body and ferritin (SLC40A1) responsible for extracellular effluent cells are controlled by Nrf2 (Agyeman et al., 2012). A study recently found that ferroptosis inducers RSL3 and ML-162 induce ER stress *via* the PKR-like ER kinase (PERK)–ATF4–SESN2 pathway and subsequently induce p62 expression. p62 further inactivates Keap1, and Nrf2 was activated by p62–Keap1 interaction, and the ARE associated with iron and antioxidant systems was increased at the same time, resulting in LIP reduction (Shin et al., 2018). Exposure of astrocytes to Fe2^+^ resulted in increased Nrf2 time- and concentration-dependent expression, whereas knockdown of Nrf2 levels by siRNA resulted in greater toxicity of Fe2^+^-induced astrocytes (Cui et al., 2016). Targeting the Nrf2–HO-1 axis has been considered as a key pathway regulating cellular inflammation and oxidative stress levels (Chen-Roetling and Regan, 2017; Shin et al., 2019; Zhou et al., 2019). Heme oxygenase (HO/HMOX1) is a cytoprotective enzyme that operates as a key rate-limiting enzyme in the process of degradation of the iron-containing molecule, heme, yielding the following by-products: CO, iron, and biliverdin (Rochette et al., 2018; Maamoun et al., 2019). In murine models of doxorubicin (DOX) and ischemia/reperfusion (I/R)-induced cardiomyopathy, administering DOX to mice induced cardiomyopathy with a rapid, systemic accumulation of non-heme iron *via* heme degradation by Nrf2-mediated upregulation of HO-1, which effect was abolished in Nrf2-deficient mice (Fang et al., 2019). In acute iron exposure, Nrf2 may protect from iron-induced injury, whereas long-term iron exposure resulted in iron accumulation, cytosolic ROS formation, and increased HO-1 (HMOX-1) mRNA expression, and this was accompanied by nuclear translocation of Nrf2 and induction of its target protein NQO1 (van Raaij et al., 2018). Accordingly, these evidences prove the hypothesized that Nrf2 can regulate intracellular iron metabolism through the Nrf2–HO-1 axis pathway to regulate ferroptosis.

During the process of ferroptosis, the mitochondrial crest was decreased or disappeared, the membrane potential was changed, and the mitochondrial outer membrane was ruptured, and this led to mitochondrial function disruption. Nrf2 is required for acute exercise-induced increases in mitochondrial biogenesis genes in skeletal muscle, energy consumption, mitochondrial volume, antioxidant activity were reduced after exercise training in mice with impaired Nrf2 expression, in muscle cells of mice, ROS- and NO-regulated mitochondrial biogenesis through the Nrf2/Nrf1-dependent pathway (Merry and Ristow, 2016). PPARγ coactivator-1α (PGC-1α) acts as a key factor and connects several regulatory cascades involved in the control of mitochondrial metabolism. PGC-1α knockout dysregulates the Nrf2-dependent mitochondrial biogenesis through the PGC-1α/p38/GSK3β/Nrf2 cascade (Navarro et al., 2017; Gureev et al., 2019). In the course of research whether and how naringenin (NGN) would be able to prevent the mitochondria-related bioenergetics and redox dysfunctions induced by methylglyoxal (MG) in the human neuroblastoma SH-SY5Y cells, researchers found that NGN caused mitochondrial protection by an Nrf2/GSH-dependent manner (de Oliveira et al., 2019). Protein kinase mammalian sterile 20-like kinase 1 (MST1) modulates inflammation *via* multiple effects; Nrf2 expression was increased after deletion of MST1, whereas silencing of Nrf2 abolished the protective effects of Mst1 deletion on nasal epithelium survival and mitochondrial homeostasis (Song et al., 2019). Therefore, Nrf2 plays a key role in mitochondrial biogenesis and function and further regulates the process of ferroptosis.

### Research Progress on Ferroptosis and Neurodegenerative Diseases

Iron is the most abundant transition metal in the brain. In the CNS, iron can participate in critical functions including mitochondrial energy transduction, enzyme catalysis, mitochondrial function, myelination, synaptic plasticity (Devos et al., 2014), and neurotransmitter synthesis and decomposition (Lane et al., 2018). The blood–brain barrier (BBB) ingests iron through transferrin on brain capillary endothelial cells, and then transports it into the cerebral cytoplasm through astrocytes or through divalent cation-binding protein (DMT1) and maintains iron an approximately saturated steady state in the brain (Belaidi and Bush, 2016), thereby maintaining the normal physiological function of the nervous system. After the formal concept of ferroptosis publishing, the researchers began to believe that ferroptosis is the main driver of neuronal death in diseases such as PD, AD, and HD (Guiney et al., 2017; Morris et al., 2018; Mi et al., 2019). In animal models of aging and neurodegenerative diseases and human anatomy studies, iron levels in the brain were found to rise to varying degrees, and it was concluded that this increase may lead to age-dependent ferroptosis (Belaidi and Bush, 2016; Buijs et al., 2017). And studies also found that chronic exposure to iron for mice caused a disorder of membrane-transport protein function and intracellular iron homeostasis and resulted in a significant increase in ROS and free radical MDA, ultimately leading to neuron and glial cell dysfunction and even neuron loss (Li L. B. et al., 2019). In summary, iron content in nerve cells may play a key role in the association of neurodegenerative diseases with ferroptosis.

### Ferroptosis in Alzheimer’s Disease

AD is the most common neurodegenerative disease characterized by neurofibrillary tangles (NFTs) composed of Tau protein. Levels of iron and ferritin (iron storage protein) in brain tissue are associated with the amount of amyloid deposition (Bulk et al., 2018; Gong et al., 2019). Deletion of hippocampal neurons and astrocyte proliferation by inducing GPX4 deletion in adult mice, this change links AD to ferroptosis (Yoo et al., 2012). α-Lipoic acid (LA), a naturally occurring enzyme cofactor with antioxidant and iron chelator, studies have found that LA can stabilize cognitive function in AD patients by blocking tau-induced iron overload, lipid peroxidation, and ferroptosis-related inflammation (Zhang Y. H. et al., 2018). Recent studies have found that trihydroxychalcone is a treatment for AD by simultaneously inhibiting Aβ_1__–__42_ aggregation and ferroptosis (Cong et al., 2019), and ferroptosis-specific inhibitor Fer-1 can inhibit the accumulation of ROS/RNS and reduce the accumulation of α-synuclein under rotenone-induced oxidative stress, then protect neuroblastoma cells SH-SY5Y (Kabiraj et al., 2015). Double-stranded RNA-dependent protein kinase (PKR) is a component of a signal transduction pathway that mediates a variety of stress signals, including oxidative stress and ER stress, and is thought to be involved in neurodegenerative diseases. The researchers found that inhibition of PKR in HT22 cells can effectively inhibit endogenous oxidative stress-induced ferroptosis and protect HT22 hippocampal neurons (Hirata et al., 2019). According to existing research, there is abundant evidence to support a causative interplay between the concerted loss of iron homeostasis and amyloid plaque formation (Peters et al., 2015). The researchers found that iron-induced amyloid precursor protein (APP) processing, neuronal signaling, and cognitive behavioral damage while using cultured primary cortical neurons and APP/PS1 AD model mice to study the mechanism of AD-related mechanisms of iron-sulfate exposure *in vitro* and *in vivo* (Becerril-Ortega et al., 2014). Iron overload increases the production of KPI-APP and amyloid β by amyloid (Becerril-Ortega et al., 2014), which promoted the AD process. Therefore, in the process of AD disease, ferroptosis may play an important role, and it is worth further exploration.

### Ferroptosis in Parkinson’s Disease

The neuropathological features of PD are the loss of catecholamine neurons in vulnerable brain regions, including substantia nigra (SN) pars compacta (SNc) and blue spot, and the PD is biochemically characterized by mitochondrial dysfunction, accumulation of iron, diminished copper content, and depleted GSH levels in these regions (Liddell and White, 2018). It was beneficial for PD when using ferroptosis inhibitor-iron chelator deferiprone for PD treatment in a randomized controlled trial (Devos et al., 2014), and deferiprone could protect SN neurons and prevent PD progression in this trial. Studies have also found that ferroptosis was triggered by activation of protein kinase alpha (PKC-alpha), which then activated ERK-protein kinase [ERK-activating kinase (MEK)] in a Ras-independent manner, and then facilitated the process of PD diseases (Do Van et al., 2016). Excessive labile iron in the SNc has become a pathognomonic hallmark of PD and leads to increased production of noxious ROS, and it is common in PD-related diseases (Moreau et al., 2018). The researchers performed quantitative susceptibility mapping (QSM) iron deposition in the SN of 44 PD patients and 31 age- and sex-matched healthy controls and PD patients were found to exhibit significantly higher magnetic sensitivity values, especially in patients at advanced stage severity, confirming that iron accumulation in SN is consistent with PD progression (An et al., 2018). So it is concluded that ferroptosis may play an important role in the progression of PD disease, and that PD has been undergoing clinical evaluation through a conservative chelation mode based on drug-mediated iron redistribution (Moreau et al., 2018), and it might be a chance to cure PD in the future. Mitochondrial damage occurs in the early stages of PD and a large body of evidence indicates that the activity of mitochondrial complex ∣ was impaired in tissues after the death of PD, and another typical feature of PD was that GSH is selectively depleted from the (Liddell and White, 2018). These characteristics are the most typical characteristics of ferroptosis, suggesting that ferroptosis may play a key role in the progression of PD disease.

### Ferroptosis in Other Neurodegenerative Diseases

Studies have found that the 4-hydroxy-2-non-enal (4-HNE) adduct (a lipid peroxidation marker) in the caudate and putamen of HD brain and the striatum of HD mice was increased (Lee et al., 2011). And in the study of the damage of dopaminergic neurons by paraquat and mancozeb, it was found that NADPH oxidase activation caused lipid peroxidation, which led to ferroptosis in SH-SY5Y cells (Hou et al., 2019). Besides, the rupture of the vessel wall in hemorrhagic stroke results in the accumulation and dissolution of iron-rich erythrocytes in the brain parenchyma and subsequent excessive presence of hemoglobin and heme iron in the extracellular environment, resulting in iron-induced lipid peroxidation and ferroptosis (DeGregorio-Rocasolano et al., 2019). Particularly, recent research found that neurons respond to ferroptotic stimuli by induction of selenoproteins, including antioxidant GSH peroxidase 4 (GPX4). A single dose of Se delivered into the brain drives antioxidant GPX4 expression, protects neurons, and improves behavior in a hemorrhagic stroke model (Alim et al., 2019). Friedreich ataxia (FRDA) is a progressive neurological and cardiac degenerative disease caused by repeated amplification of GAA in the first intron of two alleles of the FXN gene. In FRDA, the expression of the encoded protein frataxin was decreased, leading to obstruction of mitochondrial matrix iron–sulfur cluster biosynthesis, mitochondrial dysfunction, and mitochondrial iron accumulation, and finally result in increased oxidative stress. Studies have found that cells treated with ferric ammonium citrate (FAC) and L-buthionine-sulfoximine (BSO) consistently show reduced GSH-dependent peroxidase activity and increased lipid peroxidation in FRDA disease model (Cotticelli et al., 2019). When comparing the erastin in the induction of ferroptosis in neurons and HT1080 fibrosarcoma cells, it was found that selective protection of neurons by class I histone deacetylase (HDAC) inhibitors and accelerated cancer cell ferroptosis (Zille et al., 2019). Through literature review, it is speculated that in animal models of neurodegenerative diseases, depletion of long-term nuclear receptor-assisted activator (NCOA4) in the brain can worsen the neurodegenerative disease phenotype due to the further inappropriate accumulation of free iron and resulted in oxidative stress (Quiles Del Rey and Mancias, 2019), and then induced ferroptosis in nerve cells. Neurodegenerative iron accumulation (NBIA) is considered to be a neurodegenerative disease. In addition to iron accumulation, it also displays the corresponding pathological changes of tau protein in AD or synaptic nucleoprotein in PD during the NBIA process (Kruer, 2013). In conclusion, we can refer that ferroptosis may be a pathological form of neurodegenerative disease.

## Evidence for Nuclear Factor E2 Related Factor 2 Regulating Ferroptosis in Neurodegenerative Diseases

Melatonin (MLT) is a chronobiotic hormone that tightly regulates the circadian rhythms setting a biological clock in vertebrates. Meanwhile, MLT is also known to regulate fundamental cellular functions by exhibiting antioxidant, antiaging, antivenom, oncostatic, cytoprotective, and immunomodulatory activities (Hardeland, 2018). Studies found that melatonin administration can reverse nervous system harmful effects of Mn through inhibition of Keap1 and consequently, activation of the Nrf2/ARE signaling pathway (Deng et al., 2015; Ahmadi and Ashrafizadeh, 2019). Recently, research has confirmed that hemin-induced ferroptosis in platelets is mediated through ROS-driven proteasome activity and inflammasome activation, which were mitigated by MLT (NaveenKumar et al., 2019). Moreover, when studying the role of cortical astrocytes in a mouse model of cerebral hemorrhage [intracerebral hemorrhage (ICH)], researchers found that MLT activates astrocytes through PKCα/Nrf2/HO-1 signaling pathway to acquire resistance to the toxicity of hemin and resist from oxidative stress (Chen et al., 2019). Brain-derived neurotrophic factor (BDNF) has been certified playing a key role in the regulation of redox-sensitive transcription factor Nrf2 in astrocytes and metabolic cooperation between astrocytes and neurons. Stimulation by BDNF generates the signaling molecule ceramide, which activates PKCζ, leading to induction of the CK2–Nrf2 signaling axis, thereby protecting dopaminergic neurons from ferroptosis (Ishii et al., 2019). Although we hypothesize the regulation of Nrf2 in the ferroptosis process of neurodegenerative diseases, it is unclear whether the neuroprotective effect in neurodegenerative disease model is through targeting Nrf2 to suppress ferroptosis regulation.

## Conclusion and Future Prospects

As a new and unique form of programed cell death, ferroptosis has aroused great interest in scientists since it was termed in 2012. Ferroptosis is initiated by severe lipid peroxidation relying on ROS generation and intracellular iron overload, but many of its physiological effects are yet to be defined. Oxidative stress can lead to the loss of neurons in various diseases and aging processes. Two key pathways to the maintenance of redox homeostasis in the body are GSH and thioredoxin antioxidant pathways. Not only is GSH regulated by Nrf2, the thioredoxin-based antioxidant system (TXN1, TXNRD1) is also regulated by Nrf2, indicating that Nrf2 regulates the redox state of proteins (Reisman et al., 2009). With the burgeoning in ferroptosis research, numerous proteins have been shown to be involved in the regulation of ferroptosis and can roughly classify them into four categories: GPX4 synthesis and function-related, iron metabolism-related, lipid peroxidation-related, transcription factors and others. Of particular importance is the fact that the GPX4 synthesis and function, intracellular iron homeostasis, and lipid peroxidation clearing can all be mediated by Nrf2 target genes. We further verified the regulatory effect of Nrf2 on ferroptosis-related pathways through PPI network analysis and combined the existing evidence to propose the hypothesis that intracellular iron concentration is mainly regulated by the Nrf2–HO-1 pathway, although more direct evidence is needed to verify the hypothesis.

Nrf2 has also become a consensus in the regulation of neurodegenerative diseases. There is already ample evidence that ferroptosis does occur during neurodegenerative diseases, and the treatment of neurodegenerative diseases through targeting ferroptosis regulators has proven to be reliable. Therefore, targeting the antioxidant transcription factor Nrf2 to suppress ferroptosis is an attractive new option for neurodegenerative control. Even though targeting Nrf2 has been demonstrated to exert anti-ferroptosis effects in the context of cancer cells, there is still insufficient evidence in the field of neurodegenerative disease therapy, and the specific markers, genetic regulation, and other studies have not been clear. Therefore, this paper reviews the new finding in ferroptosis regulation, the relationship between Nrf2 and ferroptosis, and the research progress of ferroptosis in neurodegenerative diseases. In summary, we propose that the regulation of neuronal and neuronal ferroptosis by targeting Nrf2 is expected to become a new direction for the prevention and treatment of neurodegenerative diseases. And it is of great significance for the study of human neurological diseases and aging, especially in neurodegenerative diseases such as PD, AD, and HD.

## Author Contributions

XS and DL wrote the manuscript through a large number of literature research. DL guided the writing and reformed the manuscript. All authors contributed to the literature review, discussion, and writing of the manuscript.

## Conflict of Interest

The authors declare that the research was conducted in the absence of any commercial or financial relationships that could be construed as a potential conflict of interest.
